# Changes of knowledge and practical skills before and after retraining for basic life support: Focused on students of Dental School

**DOI:** 10.7150/ijms.47343

**Published:** 2020-10-22

**Authors:** Seo-Yoon Kim, Dongmin Shin, Hyun Jeong Kim, Myong-Hwan Karm

**Affiliations:** 1 Department of Dental Anesthesiology, School of Dentistry, Seoul National University, Seoul, Republic of Korea.; 2 Department of Emergency Medical Service, Korea National University of Transportation, Chungcheongbuk-do, Republic of Korea.; 3 Department of Dental Anesthesiology and Dental Research Institute, School of Dentistry, Seoul National University, Seoul, Republic of Korea.; 4 Department of Dental Anesthesiology, Seoul National University Dental Hospital, Seoul, Republic of Korea.

**Keywords:** American Heart Association, basic life support, cardiopulmonary resuscitation, dentistry, retraining, students

## Abstract

**Background:** Considering the increasing possibility of emergency situations in dental clinics over time, we conducted this study to evaluate the changes in the knowledge and practical skills of students of dental school before and after retraining for 2 years after the initial education on basic life support (BLS) of the American Heart Association (AHA).

**Methods:** All third-year students of dental school who had received the same education on BLS provider training of the AHA 2 years earlier were included in this study. Among them, 98 students were asked to answer a questionnaire about BLS knowledge and conduct a practical skills assessment of high-quality cardiopulmonary resuscitation using Little Anne QCPR before and after retraining.

**Results:** After retraining, the level of BLS knowledge increased in all 7 categories, and BLS performance increased in all 19 subcategories. Comparison of the QCPR numerical data items before and after retraining showed that all items after retraining met the criteria recommended by the AHA.

**Conclusion:** Students of dental school had low levels of knowledge and practical skills of BLS before retraining after 2 years from the initial education and had high levels after retraining. Therefore, BLS training must be updated periodically, and more effective education methods are required to maintain BLS knowledge and practical skills.

## Introduction

With the progression of aging, the number of acute cardiac arrests increases annually 1. Studies have reported that annually, there are 420,000 cardiac arrests in the United States and 275,000 in Europe 2, 3. In Korea, this number is approximately 30,000 per year and has been increasing progressively since 2006 1. Cardiac arrests may occur anywhere, at any time, and to anyone. Moreover, there are several causes and locations of cardiac arrests, including those occurring in dental hospitals and clinics. The major risk factor for cardiac arrest is ischemic heart disease whose prevalence increases with age 4. Due to the rising number of medically compromised and elderly patients, there is also an increase in the likelihood of a medical emergency during treatment 5. In dental care, it has been reported that increasing use of drugs and invasive procedures has led to increasing number of cardiac arrests 6. Cardiac arrests account for approximately 1% of emergencies in dentistry 6-8, which indicates the high importance of prompt and high-quality basic life support (BLS) by dentists. The key determinants of survival for patients with cardiac arrest are the timely provision of cardiopulmonary resuscitation (CPR) and defibrillation 9. When the rescuers are influenced by indecision, the time for critical care is delayed, resulting in increased mortality 10. Hence, taking the time to prepare and develop a plan for emergencies can save a life 11. Therefore, dentists should be trained and competent in BLS. In this context, previous studies have demonstrated that high quality of BLS is the most important factor in determining survival in cases of cardiopulmonary arrest 12, 13. BLS practical skills include recognition of cardiac arrest, activation of local emergency medical services, initiation of CPR, and use of an automated external defibrillator (AED).

In the United States, only dentists who have received BLS training course can practice dental care; however, such a policy is nonexistent in Korea. As it is not mandatory, the level of participation in the BLS training course is low in Korea. Moreover, although there are dentists participating in BLS training, the rate of retraining after 2 years is lower despite the recommendations of the American Heart Association (AHA). Therefore, this study was conducted to compare the knowledge and practical skills competency before and after BLS retraining in students who received BLS education 2 years earlier.

## Methods

### Study design

A nonrandomized quasi-experimental design (one group pretest-posttest) was used in this study. This design examined the effectiveness of BLS training on the knowledge and practical skills of students of dental school. This study was conducted at the Department of Dental Anesthesiology, Seoul National University School of Dentistry. Approval for conducting the study was obtained from the Institutional Review Board (approval number: S-D20190023), and written informed consent was obtained from each subject who participated in the study. All aspects of subject privacy and confidentiality were preserved. This study was registered with the Clinical Research Information Service (cris.nih.go.kr/KCT0004703) and conducted according to the Declaration of Helsinki.

### Study participants

Inclusion criteria were as follows: 1) third-year students of the dental school, Seoul National University, 2) attended the AHA BLS 2015 version of the training with the same instructor 2 years earlier, and 3) agreed to both the survey and the skill test before and after retraining.

Exclusion criteria were as follows: 1) those who had received additional AHA BLS training within 2 years, 2) those who had previous experience of CPR within 2 years, 3) those who were physically inadequate for BLS training (e.g., injuries or splints of arms or legs, back pain), 4) those who refused to participate in the study, and 5) those considered as inappropriate by the researcher to participate in the study.

### BLS training

All third-year students of the dental school had received BLS education (2015 version) of the AHA from March 1, 2019 to July 15, 2020 14, 15. The training was conducted in 18 teams of 6-10 students each. The BLS training included both theoretical and practical components.

#### Theoretical training

In the first session theoretical training was conducted through a 2-h video (BLS DVD 2015, AHA, USA) with discussion.

#### Practical skills training

The second session of the training included practical training on BLS. It was repeated until the students could successfully perform every step of BLS. Practice and assessment were conducted using Little Anne QCPR^®^ (Laerdal, Norway). To prevent bias, both theoretical and practical training was provided by the same instructor (leader instructor, M.-H.K.) who was specialized and certified by the AHA had provided more than 50 BLS educational sessions. Teams with more than 6 students received practical skills training from an additional instructor (S.-Y.K.).

### Data collection

#### Pre-retraining assessment

##### Questionnaire on BLS knowledge

Before retraining, the students were asked to fill out the questionnaire addressing the knowledge of BLS. The original questionnaire is presented in the supplementary materials, and the survey items are as follows (**Table 1**).

#### Assessment of BLS practical skills

##### Assessment items

After the survey, an assessment was conducted to evaluate the effectiveness of BLS with manikins using a feedback device. The assessment involved two cycles of chest compressions and rescue breath, followed by 5 min from the initial patient assessment to deliver an electric shock using an AED. This was the same assessment sequence and items used in the BLS training course based on the 2015 AHA guidelines (**Table 2**).

##### Assessment device

Objective assessments were conducted using “Little Anne QCPR^®^” and “QCPR Training” application that can monitor in real time and save the results. The feedback device is placed inside the manikin and can connect to the instructor's tablet PC or smart phone. Numerical data such as chest compression rate, chest recoil rate, average values of chest compression depth and speed, and adequate rate of rescue breath (using pocket mask) were saved to the “CPR” version in the app. The data were saved as the name initial of students, date, and time. Other items such as CPR sequence, hand placement of chest compression, and chest compression interruption time were evaluated by the instructor as appropriate or not.

### Post-retraining assessment

#### Questionnaire on BLS knowledge

After the retraining, the students were asked to fill out the same questionnaire as done before the BLS retraining.

#### Assessment of BLS practical skills

After the completion of retraining, each student was asked to perform every step of BLS. The same assessment as done before retraining was repeated. The instructors evaluated the students' practical skills using QCPR.

### Statistical analysis

Frequency analysis and descriptive statistics analysis were conducted to compare the assessment results of knowledge and practical skills of BLS before and after retraining. Frequency and McNemar's tests were analyzed for the number of responses and the results of each option. In cases of correct answer, 1 point was given, 0 points were assigned for a wrong answer, and the total score before and after retraining was compared by a paired *t*-test. The results of BLS skills assessment before and after retraining were compared using McNemar's test, paired *t*-test, and Wilcoxon signed-rank test. All statistical analyses were conducted using IBS SPSS Statistics version 21 (SPSS, Inc., Chicago, IL, USA), with *p* < 0.05 being considered as statistically significant.

## Results

### Demographic characteristics

A total of 98 participants fulfilled the inclusion and exclusion criteria. Regarding the demographic characteristics, there were 54 men (55.1%) and 44 women (44.9%) with a mean age of 25.7 ± 1.8 years.

### Knowledge before and after retraining

**Figure 1:**
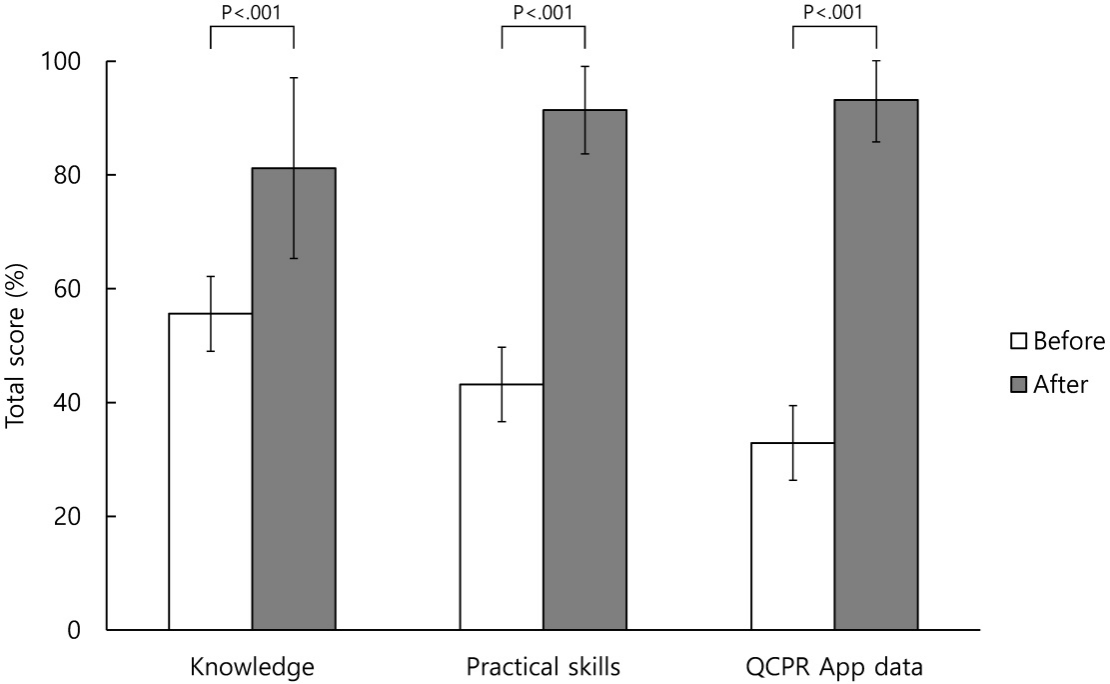
Total score of before and after basic life support retraining.

**Table 3** shows the correct answers to the knowledge questionnaire items before and after BLS training. There was an increase in the percentage of correct answers regarding BLS knowledge for all items after retraining, with a statistically significant difference (*p* < 0.01) being observed for all the items (**Table 3**). The total score of BLS knowledge increased from 55.6 ± 16.7 to 81.2 ± 15.9 points when converted into a 100-point scale before and after retraining and was statistically significant (*p* < 0.001) (**Figure 1**).

### Practical skills before and after retraining

The proportions of appropriate ability to practical skills items before and after BLS training are presented in **Table 4.** The percentage of appropriate ability about BLS practical skills increased for all items after retraining. A statistically significant difference (*p* < 0.05) was observed in 16 of these items (**Table 4**). In the “patient assessment” part, the statistically significant items were “check breathing,” “check pulse,” and “shout for nearby help and active the EMS system / obtain an AED.” In the “chest compression” part, all of 7 items were statistically significant. In the “ventilation” part, the statistically significant items were “adequate ventilation” and “number of ventilations.” In the “using an AED” part, the statistically significant items were “attach the pad correctly,” “clear from the patient for analysis,” “clear from the patient for shock delivery,” and “deliver shock and continue chest compression.” The total score of BLS practical skills increased from 43.2 ± 11.0 to 91.4 ± 7.7 points when converted into a 100-point scale before and after retraining and was statistically significant (*p* < 0.001) (**Figure 1**).

### Comparison of BLS skills numerical data using QCPR

**Figure 2:**
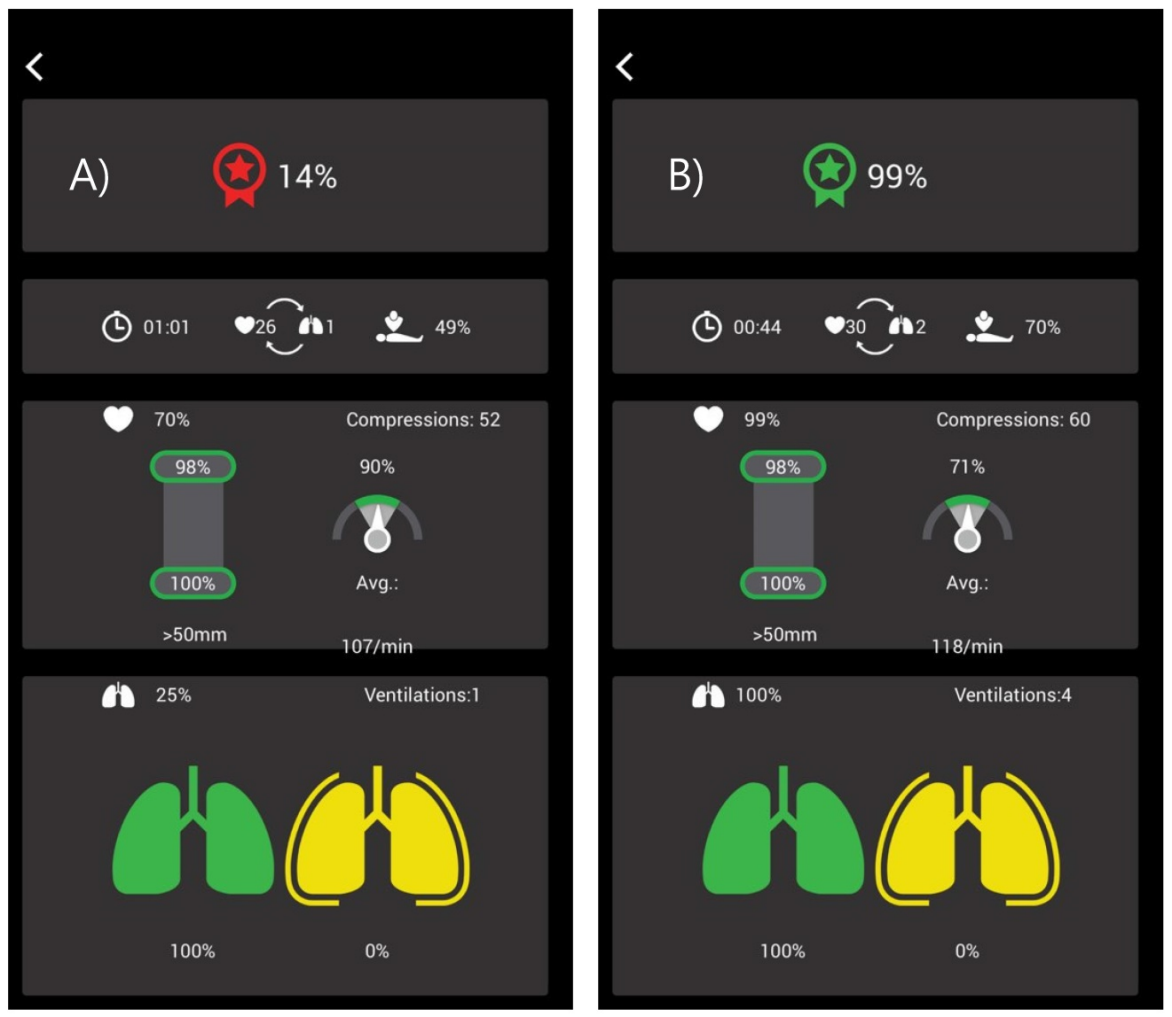
QCPR App data. The figure is the real QCPR App data of a dental school student. Before retraining, the total basic life support skill score was 14% and it improved to 99% after retraining.

**Figure 2** shows the actual data of a student before and after retraining. Before training, 9 of 10 subcategories did not meet the guideline targets; after training, all the items almost met the guidelines. Statistically significant differences (*p* < 0.05) were observed among the 9 items (**Table 5**). In the “chest compression” part, the statistically significant items were “compression with adequate speed rate,” “average depth,” “compression with adequate depth rate,” “flow fraction,” and “chest compression total score.” In the “ventilation” part, the statistically significant items were “average speed,” “adequate ventilation,” “number of ventilations in 2 cycles,” and “ventilation total score”.

In the “ventilation” part, the statistically significant items were “adequate ventilation,” “number of ventilations in 2 cycles,” and “ventilation total score.” The total scores for chest compression, ventilation, and all procedures of BLS increased from 32.9 ± 23.7 to 93.2 ± 7.4 points after retraining and exhibited statistically significant differences (*p* < 0.001) (**Figure 1**).

## Discussion

Before retraining, the levels of knowledge and practical skills of the students were extremely low. Previous studies have also similarly showed that dental students' knowledge of BLS was very poor before training 16-19. The present results reveal that the educational effect was not maintained appropriately after 2 years due to lack of retraining and less real-time experience of cardiac arrest. After retraining, the knowledge level was improved to 81.2 ± 15.9 points, and the total practical skills score was 91.4 ± 7.7 points. This finding indicates that BLS training improved the knowledge and practical skills related to BLS.

According to the recently revised 2015 BLS guidelines of the AHA, complete retraining within 2 years after initial training is recommended to maintain the effectiveness of basic resuscitation training to 9. Several studies have suggested that healthcare providers who do not frequently perform CPR, such as dentists and students of dental school, could rapidly forget the knowledge and skills acquired during education within 3-6 months after the completion of the BLS course 20, 21. In a previous study, Seo et al. evaluated the CPR knowledge and practical skills of first- and fourth-year dental school students before receiving CPR education and compared the correct answer rate according to previous education experience 22. They observed no statistically significant differences and further found that the 3-year retraining interval was not maintained in terms of CPR knowledge and performance. In the present study, we observed that BLS knowledge and practical skills were not maintained even with 2-year retraining interval.

The present study demonstrated high knowledge and practical skills after retraining. The average total score of BLS knowledge was 78.5 when converted into a 100-point scale after retraining, and a statistically significant difference was found when compared to that before retraining. However, the percentages of correct answers regarding “hand placement of chest compression” and “appropriate depth of chest compression” were low although the students were retrained. According to recent guidelines, proper hand placement for chest compression is “on the lower half of the sternum,” and the proper depth of chest compression is “more than 50 mm but not more than 60 mm.” The most inaccurate answers for these items were “centered between the nipples” and “more than 50 mm,” respectively. These aspects should be further emphasized during the training. Moreover, the percentage of correct answer of “recently revised guidelines year” question revealed some increasing trend after retraining, but all scores were low. This signifies that students do not keep up with guideline revisions every 5 years. Currently, the AHA revises its guidelines every 5 years based on the latest clinical research and recommend the use of updated guidelines. In fact, every 1 min of delay in cardiac arrest reduces the probability of survival by 7%-10% 23. Therefore, CPR standards have been extensively revised to shorten the time required to perform high-quality CPR after the recognition of cardiac arrest 24-26.

Work in the field of dentistry is fraught with risks leading to life-threatening emergencies because dental procedures are performed in the oral cavity, which essentially involves breathing. Negligence of dentists in the case of death of patients during dental care is one of the leading causes of dental legal litigation worldwide 27. A study analyzing the precedents of medical disputes in the dental field in Korea reported that this rate was the second highest among all medical disputes 28. Considering these legal issues, every dentist must be familiar with various protocols (such as BLS) to efficiently manage the emergencies arising in the dental office. They should also be aware of the revision cycle of BLS guidelines, which should be emphasized in BLS training.

In the present study, the average score of BLS practical skills ability was 91.4 points, which was very appropriate. This result was consistent with the results of studies concerning retraining CPR in similar groups 18, 19, 22, 29, 30. A previous study of medical students reported a significant increase in the total score of the retraining group between 18 and 23 months 31. Another study reported that nursing students maintained the ability to compress to the appropriate depth until 12 months after the initial monthly retraining 32. The reason for the large difference in scores before and after retraining in the present study might be that students could concentrate more in class according to recognize themselves did not perform properly, and practice repetitively during retraining. Moreover, using the feedback devices to practice during retraining might have affected this outcome.

The 2015 AHA guidelines recommend the use of feedback devices in CPR training 9. This has been demonstrated to improve CPR outcomes in several studies. For instance, distributed CPR training using manikins with real-time visual feedback demonstrated excellent CPR performance with >90% accurate compression depth (50-60 mm) and speed (100-120/min) at 1, 3, 6, and 12 months 33, 34. Similarly, Brendan et al. demonstrated that the group receiving objective feedback showed significant improvement in the acquisition and retention of chest compression skills 6 weeks after the initial training compared with those who did not receive feedback using the “Resusci^®^ Anne Skillreporter” manikin 35.

One of the obvious differences between continuing BLS training compared with the first training year (2017) is the use of feedback devices during BLS training in our study. In 2017, students did not use feedback devices and received feedback based on the instructor. However, all students received equal feedback during retraining using the skill guide, a feedback device that was directly connected to the QCPR manikin to measure CPR skills. This suggests the presence of a difference in the degree of education acquisition and self-assessment in education, which was related to the maintenance of the educational effect, as reported in the other abovementioned studies. During retraining, students can assess their own skills in real time using feedback. Satisfaction survey results demonstrated that students were satisfied with their improvement after feedback through the skill guide. Wik et al. asserted that the effects of educating students through the use of automatic voice-directed manikins to feedback their own CPR techniques persisted after 6-12 months. It would be desirable to use a feedback device for effective and retentive BLS training 36, 37.

The results of the present study showed that levels of knowledge and practical skills were low and the effect of BLS training was not maintained 2 years later. Therefore, retraining should be performed more often than the current 2-year cycle. Winchell et al. reported limited effect after 6 months of CPR training 38, and Kang et al. insisted on reducing the retraining period to 2 years 39. Hence, more research on retraining interval to maintain the effect of BLS training is necessary, and student training should be conducted at appropriate time intervals. However, providing BLS retraining to all healthcare providers would be difficult with a shortened training cycle. In this regard, we must consider other methods to maintain the effects of BLS training. We believe that it is necessary to identify methods to not only shorten the period of retraining but also maintain the effectiveness of BLS education. Einspruch et al. demonstrated that the group trained by video self-instruction showed significantly superior CPR performance both immediately after training and 2 months after training compared with the group trained using the existing Heartsaver method 40. In addition, Castillo et al. reported that the group trained using a combination of a self-training video, a new website, a Moodle platform, an intelligent manikin and 45 min of instructor presentation showed the same or higher performance than the control group (face-to-face training based on the European Resuscitation Council Guidelines) in terms of the knowledge and skills 6 months later 41. It is expected that these various teaching methods can help improve and maintain BLS knowledge and skills. Therefore, more research is required to develop methods and training programs to improve the knowledge and practical skills of BLS.

There were several limitations in the present study that need to be considered. First, the study design is restrictive (a single group, pretest and posttest). It was conducted with a non-blind design. However, this should not have affected the results because the variables of assessment were objective. Moreover, this study was conducted at only one dental school, due to which generalization of the findings was not possible. Nevertheless, this study contributes to a learning approach of students of dental school and dentists. The second limitation is the sample size. Although we conducted a pilot study to determine the sample size, the calculated sample size was less than 5 because the difference between before and after retraining was clear. Therefore, we planned to conduct the present study for students of dental school in a grade. Third, pediatric and infant BLS assessments were not conducted. As BLS includes education on adults, children, and infants, further research on children and infants would be necessary. Fourth, the same instructor presented the same BLS training without a feedback device in the original training. As feedback devices have been found to improve the quality of BLS training, if we had been training with feedback devices before 2 years, the pre-retraining results might have been a little better. Nonetheless, despite these limitations, the present study provides important information about an objective evaluation of pre- and post-retraining results for students of dental school.

## Conclusion

This study confirmed that BLS knowledge and practical skills decreased significantly 2 years after BLS training. Therefore, it is necessary to update BLS training periodically and also implement more effective education methods to maintain BLS knowledge and practical skills.

## Supplementary Material

Supplementary table S1.

## Figures and Tables

**Table 1:** Questionnarie about basic life support knowledge

No	Questions	Answers (ex)
1	Chest compression to respiration rate	30:2
2	Steps of CPR revised in 2015	Check responsiveness → Shout for nearby help → Check breathing & pulse, Active the EMS system, get an AED → Provide chest compression
3	Chest compression depth & speed	(1) Depth: 50-60 mm (2) Speed: 100-120/min
4	Hand placement of chest compression	On the lower half of the sternum
5	Airway management	Head tilt & Chin lift
6	How to use an AED	Turn on the power → Attach the pad → Clear from the patient for analysis → Analyze CPR rhythm → Clear from the patient for shock delivery → Deliver shock → Continue chest compression.
7	Year of most recent AHA guideline revision	2015

AED, automatic external defibrillator; AHA, American Heart Association; CPR, cardiopulmonary resuscitation; EMS, emergency medical service.

**Table 2:** Highlights of basic life support (2015)

No.	Contents
1	CPR sequence
2	Chest compression speed
3	Chest compression depth
4	Chest recoil rate
5	Chest compression interruption time
6	Airway management
7	Compression and rescue breath ratio
8	Hand placement of chest compression

CPR, cardiopulmonary resuscitation.

**Table 3:** Comparison of correct answers of basic life support knowledge questionnaire before and after basic life support retraining

BLS component (n = 98)	Before	After	*p*-value
Chest compression to respiration rate	69 (70.4)	98 (100)	<0.001
Steps of CPR revised in 2015	82 (83.7)	97 (99.0)	<0.001
Chest compression position	10 (10.2)	66 (67.3)	<0.001
Chest compression depth & speed	50 (51.0)	68 (69.4)	0.010
Airway management	59 (60.2)	76 (77.6)	0.001
How to use an AED	71 (72.4)	92 (93.9)	<0.001
AHA guidelines revised recently	41 (41.8)	60 (61.2)	0.009

Values give as number (%); AED, automatic external defibrillator; AHA, American Heart Association; CPR, cardiopulmonary resuscitation.

**Table 4:** Comparison of basic life support skills before and after retraining (n = 98)

BLS component	Behavior	Before	After	*p*-value
Patient assessment	Check responsiveness	97 (99.0)	98 (100)	1
	Check breathing	32 (32.7)	98 (100)	<0.001
	Check pulse within 5~10 sec	26 (26.5)	98 (100)	<0.001
	Shout for nearby help and active the EMS system/obtain an AED	55 (56.1)	98 (100)	<0.001
Chest compression	Compression:ventilation ratio for adults (30:2)	7 (7.1)	83 (84.7)	<0.001
	Hand placement on the lower half of the sternum	12 (12.2)	98 (100)	<0.001
	Average compression depth to 50-60 mm*	18 (39.1)	37 (80.4)	<0.001
	Average compression rate of 100-120/min	59 (60.2)	93 (94.9)	<0.001
	Complete chest recoil	61 (62.2)	74 (75.5)	0.026
	Minimizing interruptions between chest compression (<10sec)	8 (8.2)	96 (98.0)	<0.001
	Number of compressions in 2 cycles	41 (41.8)	78 (79.6)	<0.001
Ventilation	Adequate ventilation	22 (22.4)	88 (89.8)	<0.001
	Hyperventilation	79 (80.6)	88 (89.8)	0.108
	Number of ventilations in 2 cycles	10 (10.2)	85 (86.7)	<0.001
Using an AED	Turn on the power	96 (98.0)	98 (100)	0.500
	Attach the pad correctly	78 (79.6)	98 (100)	<0.001
	Clear from the patient for analysis	23 (23.5)	98 (100)	<0.001
	Clear from the patient for shock delivery	16 (16.3)	98 (100)	<0.001
	Deliver shock and continue chest compression	65 (66.3)	98 (100)	<0.001

Values are presented as number (%); AED, automatic external defibrillator; BLS, basic life support; EMS, emergency medical service. *There are missing data; the number of participants was 46.

**Table 5:** Comparison of basic life support skills numerical data using QCPR

BLS component	Behavior	Guideline target	Before	After	*p*-value
Chest compression	Average speed (n/min)	100-120	116.2 ± 10.4	112.9 ± 4.9	0.006
	Compression with adequate speed rate (%)	100	46.4 ± 31.5	83.5 ± 18.5	<0.001
	Average depth (mm)	50-60	60.6 ± 11.5	56.2 ± 11.7	0.019
	Compression with adequate depth rate (%)	100	91.1 ± 19.1	98.9 ± 4.2	<0.001
	Compressions fully released (%)	100	93.5 ± 18.5	96.8 ± 9.6	0.065
	Flow fraction (%)	60-100	50.5 ± 11.4	66.3 ± 5.3	<0.001
	Number of compressions in 2 cycles	60	61.0 ± 9.5	61.1 ± 5.8	0.908
	Chest compression total score	100	78.0 ± 22.9	96.4 ± 6.3	<0.001
Ventilation	Adequate ventilation (%)	100	30.9 ± 42.4	93.6 ± 21.9	<0.001
	Hyperventilation (%)	0	10.9 ± 24.9	6.3 ± 21.9	0.174
	Number of ventilations in 2 cycles	4	1.2 ± 1.7	4.0 ± 0.6	<0.001
	Ventilation total score	100	25.5 ± 35.6	94.7 ± 13.9	<0.001

Values are presented as mean ± SD; BLS, basic life support.

## Abbreviations

BLS: basic life support
AHA: American Heart Association
CPR: cardiopulmonary resuscitation
AED: automated external defibrillator
EMS: emergency medical service
