# Supplementary material for: Changes of knowledge and practical skills before and after retraining for basic life support: Focused on students of Dental School
Source: Int J Med Sci. 2020 Oct 22;17(18):3082–90. doi: 10.7150/ijms.47343 (PMC7646099; doi:10.7150/ijms.47343)
Supplement: Supplementary file 1 — Supplementary table S1. [file ijmsv17p3082s1.pdf]

Supplementary materials

Table S1. Questionnaires

No	Questionnaire
**1**	**Please write your gender and age.**<br>- Gender: (Male / Female)<br>- Age: ( ) years old
**2**	**Please write your name initials.**<br>[3 characters in uppercase alphabetical order, last name, first name (e.g., Hong Gil-dong → HGD)]<br>- ( )
**3**	**Have you ever taken this American Heart Association Basic Life Support Provider course?**<br>1) Yes 2) No (Go to question 5)
**4**	**When did you take a previous training?**<br>1) 2019 2) 2018 3) 2017 4) 2016 5) Before 2016
**5**	**What is the ratio of chest compressions to rescue breath during cardiopulmonary resuscitation? (in adults)**<br>1) 30: 2 2) 30: 1 3) 15: 2 4) 15: 1
**6**	**What are the steps of CPR?**<br>1) Check responsiveness → Shout for nearby help → Check breathing & pulse,

	Activate the EMS system and obtain an AED → Provide chest compressions<br>2) Shout for nearby help → Check responsiveness → Check breathing & pulse → Activate the EMS system and obtain an AED → Provide chest compression<br>3) Shout for nearby help → Activate the EMS system and obtain an AED → Check responsiveness → Check breathing & pulse → Provide chest compressions
**7**	**Which is the right location for chest compression?**<br>1) In the middle of the chest between the nipples<br>2) On the upper half of the sternum<br>3) On the lower half of the sternum<br>4) Central point of the sternum
**8**	**What is the proper depth and speed of chest compression? (in adults)**<br>1) 50 mm or more, 100 times/minute or more<br>2) 50 mm or more, 100–120 times/minute<br>3) 50–60 mm, more than 100 times/minute<br>4) 50–60 mm, 100–120 times/minute
**9**	**How do you manage the airway manually?**<br>1) Raise the chin<br>2) Turn the head sideways<br>3) Support the neck with a pillow<br>4) Tilt the head and chin
**10**	**What is the sequence of using an AED?**

	[Turn on the power → Attach the Pad → (a) → Clear from the patient → (b) → (c) → Deliver electric shock → Continue chest compression]<br>1) Connect the cord, Clear from the patient, Analyze the CPR rhythm<br>2) Analyze the CPR rhythm, Connect the cord, Clear from the patient<br>3) Analyze the CPR rhythm, Deliver electric shock, Clear from the patient<br>4) Connect the cord, Analyze the CPR rhythm, Clear from the patient
**11**	**When was the most recent revised AHA CPR guideline released?**<br>1) 2010 2) 2013 3) 2015 4) 2017 5) 2019

AED = automatic external defibrillator; AHA = American Heart Association; CPR = cardiopulmonary resuscitation; EMS = emergency medical system. There is no difference in importance for each questionnaire item.
